# Assessment of drug related problems among type 2 diabetes mellitus patients with hypertension in Hiwot Fana Specialized University Hospital, Harar, Eastern Ethiopia

**DOI:** 10.1186/s13104-018-3838-z

**Published:** 2018-10-12

**Authors:** Yohanes Ayele, Kibkab Melaku, Mesay Dechasa, Mohammed Biset Ayalew, Boressa Adugna Horsa

**Affiliations:** 1 Hiwot Fana Specialized University Hospital, Harar, Ethiopia; 2 Department of Clinical Pharmacy, School of Pharmacy, College of Health and Medical Sciences, Haramaya University, P.O.Box 235, Harar, Ethiopia; 3 Department of Clinical Pharmacy, School of Pharmacy, College of Medicine and Health Sciences, University of Gondar, Gondar, Ethiopia

**Keywords:** Drug related problems, Type 2 diabetic mellitus, Hypertension

## Abstract

**Objective:**

This study was conducted to assess magnitude and pattern of drug related problems among patients with type 2 diabetes mellitus (T2DM) and hypertension.

**Results:**

This study identified 364 drug related problems (DRPs) across the three categories of drug related problems, giving an average of 1.8 DRPs per patient. The effect of drug treatment being not optimal 179 (49.2%), untreated indication and symptoms 77 (21.1%), unnecessary drug-treatment 39 (10.7%) and adverse drug reactions 69 (19%) were the most frequent categories of DRPs identified. In general, high prevalence of drug-related problems was identified among patients with T2DM hypertension. The effect of drug treatment being not optimal, untreated indication and symptoms, unnecessary drug-treatment and adverse drug reactions were the most frequent categories of drug related problems identified. Therefore, the clinicians should work to improve patient care through prevention and resolving drug related problems since it can affect the quality of the care significantly.

## Introduction

Diabetes mellitus is a group of metabolic disorders caused either by the inability to produce insulin or by the body not being able to use insulin effectively or both [1]. Globally, 415 million adults are estimated to have diabetes currently and there are 318 million adults with impaired glucose tolerance which puts them at high risk of developing the disease in the future. Developing countries is suffering from increasing burden of diabetes mellitus [1, 2]. In 2015, it was estimated that 9.5–29.3 million peoples live with diabetes in the Africa Region [3].

American diabetic association classification categorizes diabetes into four categories; type 1, type 2, gestational and other diabetes [4]. T2DM accounts for 90–95% of the incidence of diabetes and is associated with a strong genetic predisposition as well as age, obesity and lack of physical activity [5]. Hypertension is a common comorbidity in T2DM patients with a prevalence of up to two-thirds of the population and it may be present by the time T2DM is diagnosed or even before the onset of hyperglycaemia [6]. The coexistence of hypertension and T2DM often indicates more severe disease and increases risk of macro vascular and microvascular complications [7–9].

Effective diabetes management often presents enormous challenges. Not surprisingly, clinicians and patients can be overwhelmed by the need to address comorbid chronic conditions in addition to patients’ diabetes-specific treatment goals. Furthermore, during management undesirable event which involves or is suspected to relate to drug therapy may occur, which is often termed as DRPs [10, 11].

DRPs could prevent or delay patients from achieving desired therapeutic goals and it has been attributed to unnecessary over prescription of drugs, substantial worsening of diseases, avoidable increases in hospital admission rates, and longer hospital stays leading to a significant medical burden [12, 13]. However, DRPs are preventable in most cases [14]. Hence understanding the magnitude of the DRPs and pattern of its occurrence would be important input for the practitioner in the process of DM management particularly in the patients with coexisting disease. Therefore, this study was conducted to assess the prevalence and pattern of DRPs among patients with T2DM and Hypertension in Hiwot Fana Specialized University Hospital (HFSUH), Eastern Ethiopia.

## Main text

### Methods

#### Study design and settings

Institution based retrospective cross-sectional study was conducted in HFSUH in Harar city from May to 30 August, 2017.

#### Study population

All diabetic patients on chronic follow up at HFSUH were the source populations. All adult (> 18 years) patients with T2DM and hypertension and on follow up during data collection period were study populations. Patients with missing or incomplete data were excluded.

#### Sample size determination and sampling technique

The actual sample size for the study was determined using the formula for single population proportion. From HFSUH data the number of diabetic patient uses as follow up in HFSUH was 356, the minimum sample size required for study was determined to 203 individuals.

#### Data collection, processing and analysis

The record of patients with types 2 diabetes and hypertension and currently on follow up was identified until the targeted sample size was achieved. The data was collected using data extraction format. The format was designed to collect information on sociodemographic characteristics of patients, disease characteristics of patients and medications. The collected data was revised for its completeness and consistency before it was entered into epi-data and processed and analyzed using SPSS version 20. The findings were presented using summary measures and displayed using tables.

#### Evaluation of drug related problems and classification

There have been different approaches to classify and summarize DRPs. However, few of these systems are validated and reliable [15]. We used the Pharmaceutical Care Network Europe Classification scheme for drug-related problems V8.02 [13]. Identification of DRPs was assessed by two clinical pharmacists who were trained on study protocol developed based on the Drug Information Handbook [16], standards of medical care in diabetes 2017 [17] and Joint National Committee (JNC) 8 [18].

#### Operational definition

Nephropathy: Presence of albuminuria which defined as urine albumin to-creatinine ratio ≥ 30 mg/g creatinine measured in two of three specimens collected within a 3- to 6-month period [4].

Retinopathy: Presence of clinically significant macular edema, severe non-proliferative or proliferative retinopathy seen under high-quality fundus photographs [4].

Neuropathy: Diverse clinical manifestations including diabetic peripheral neuropathy, autonomic neuropathy, cardiovascular autonomic neuropathy, and gastrointestinal neuropathies [4].

### Results

#### Socio-demographic characteristics of the Patients

A total of 203 patients were included in this study out of which 107 (52.7%) were female and 96 (47.3%) were male. The majority 137 (67.5) patients’ lives in urban area and 139 (68.5%) patients are married (Table 1).

**Table 1:** The demographic characteristic of patients with T2DM and HTN in Hiwot Fana Specialized University Hospital, 2017 (n = 203)

Variables	n (%)
Sex	
Male	96 (47.3)
Female	107 (52.7)
Age	
> 50	158 (77.8)
≤ 50	45 (22.2)
Residents	
Urban	137 (67.5)
Rural	66 (32.5)
Marital status	
Married	139 (68.5)
Single	64 (31.5)
Smoking	
Non-smoker	113 (55.7)
Smoker	90 (44.3)
Alcohol consumption	
Yes	63 (31.0)
No	140 (69.0)

#### Disease characteristics of patients

More than half 109 (53.7%) of patients’ lived with diabetes for 6–10 years, 62 (30.5%) lived with diabetes for less than 5 years. The mean T2DM duration was 7.4 years. Regarding to fasting blood sugar, a large portion (43.3%) of the patients 88 had blood glucose level of greater than 180 mg/dL in their last visit. Concerning to HTN, the BP level at last visit were less than or equal to 140/80 for 138 (67.9%) patients whereas 65 (32.1%) patient had BP above 140/80 mmHg. In this study, 122 (60.1%) patients have comorbidity other than HTN. Moreover, about 71% of patients developed T2DM related complications. The most prevalent T2DM related complication were Neuropathy 45 (22.2%) followed by Retinopathy 25 (12.3%) (Table 2).

**Table 2:** The disease characteristic of patients with T2DM and HTN in Hiwot Fana Specialized University Hospital, 2017 (n = 203)

Disease characteristics	n (%)
Duration of DM (years)	
< 5	62 (30.5)
≥ 5–10	109 (53.7)
≥ 10	32 (15.8)
FBS at last visit (mg/dL)	
> 180	88 (43.3)
140–180	45 (22.2)
< 140	68 (33.5)
BP at last visit	
≤ 140/80	138 (67.9)
> 140/80	65 (32.1)
Comorbidity other than HTN (n = 122)	
Ischemic heart disease (IHD)	47 (38.6)
Dyslipidaemia	31 (25.4)
Heart failure	18 (14.7)
Peripheral arterial disease	14 (11.5)
Other*	12 (9.8)
T2DM complications (n = 144)	
Neuropathy	45 (31.2)
Neuropathy and retinopathy	31 (21.5)
Retinopathy	25 (17.4)
Nephropathy	23 (16.0)
Diabetic foot ulcer	20 (13.9)

*FBS* fasting blood sugar, *BP* blood pressure

^a^Urinary tract infection, chronic obstructive pulmonary disease, acute pyelonephritis, portal HTN

#### Medications used in patients with type 2 diabetes mellitus and hypertension

In this study, 93 (45.8%) patients received monotherapy out of which 70 (34.5%) were metformin, 16 (7.9%) insulin and 7 (3.4%) glibenclamide. More than half of the patients received dual therapy; metformin and glibenclamide 21 (19%), insulin and metformin 21 (10.3%) and insulin and glibenclamide 11 (5.4%). Regarding HTN management, 160 (78.8%) patients received medication for hypertension. A large share of patients 93 (58.1%) received mono therapy. The angiotensin converting enzyme inhibitors (ACEIs) particularly enalapril were the most frequently used drug followed by the calcium channel blockers (CCBs) (Table 3).

**Table 3:** The characteristics of medications used to manage patients with T2DM and HTN in Hiwot Fana Specialized University Hospital, 2017 (n = 203)

Medication characteristics	n (%)
DM medication	
Metformin + glibenclamide	78 (38.4)
Metformin	70 (34.5)
Insulin + metformin	21 (10.3)
Insulin	16 (7.9)
Insulin + glibenclamide	11 (5.4)
Glibenclamide	7 (3.4)
Classes of HTN medication used	
ACEIs	41 (20.2)
CCBs	29 (14.3)
Diuretics	15 (7.4)
Beta blockers	4 (2.0)
Centrally acting α_2_ agonist	4 (2.0)
Combined classes	67 (33.0)
ADR reported	
Gastrointestinal upset	36 (17.7)
Dry cough	24 (11.8)
Hypoglycaemia	7 (3.4)
Ankle oedema	2 (1.0)

*ACEIs* angiotensin converting enzyme inhibitors, *CCBs* calcium channel blockers

#### Drug related problems in type 2 diabetic with hypertension patients

In this study, 364 DRPs were identified which is about 1.8 DRPs per patient. Among this, 179 (49.2%) patients were not on optimal drug treatment. It was found that 25 stage II hypertensive patients received monotherapy whereas 133 patients did not achieve their glycaemic goal. Seventy-seven patients did not get drug therapy for a clear indications and symptoms. Forty-two T2DM complications were not treated and 29 hypertensive patients did not receive the antihypertensive therapy. In 39 (10.7%) cases, there was unnecessary therapy. Fourteen stage I hypertensive patients received dual therapy while the appropriate therapy should have been a mono therapy. Sixty-nine cases of adverse reactions were reported and antidiabetic drugs were associated with 43 of all the cases.

### Discussion

In current study, 364 DRPs were recorded which is about 1.8 DRPs per patient. This finding is lower than study conducted among Jordan [19] ambulatory patients where 11.2 DRPs per patient were reported but higher than study conducted in Nigeria [20] and southern Ethiopia [21]. The large variation seen on magnitude of DRPs across studies might be due to different classification of system used to classify DRPs and settings in which DRPs were assessed. Despite the difference seen in magnitude of DRPs, the finding in our study shows the need to make intervention on the DRPs so that the patients improve success of treatment outcomes. Furthermore we recommend future studies to use similar DRPs classification system to generate comparable evidences.

For patients with diabetes and HTN a target reduction of BP of ≤ 130/80 mmHg is recommended [4]. Achieving this target is often challenging and patients need one or more drugs to achieve this goal. The poor BP control reported in this study might be explained by the patients not receiving optimal therapy. This result is in agreement with study conducted in Jordan [19] and Southern Ethiopia [21] where patient being not in optimal therapy was most commonly observed DRP.

Similarly, the American diabetic association (ADA) guideline recommends Pre-prandial plasma glucose target goal of l80–130 mg/dL is although the stringency varies based on the patient factors. In this study, 133 T2DM patients did not achieve the desired glycaemic goal. Although poor glycaemic control could be attributed to different factors such as life style modification, presence of other comorbidities; DRPs particularly non-adherence could be a major factor in this population. This in turn could be related to forgetfulness, refusal to take medication, cost of medication and unavailability [22, 23]. Hence the health care providers should consider the issue of adherence to medication as important component T2DM patients’ management.

The second most frequently observed DRP in present study is untreated symptoms and indication. Evidence indicates that patients with T2DM and HTN require aggressive medication therapy in order to halt and prevent morbidity, mortality and unnecessary hospital stay and disability [4, 18]. However, in this study 28 stage I hypertensive patients’ and one stage II hypertensive patient did not receives antihypertensive therapy. Moreover, there were also other comorbidities and DM related complications which were not given drug therapy.

In agreement with study conducted in other settings [24, 25], use of unnecessary drugs is commonly observed DRPs. In this study, 39 patients received unnecessary medication therapy for a clinical condition in which a single therapy suffice or for the condition that is not existed. Some drugs were prescribed without any clear indication. These add on medications should be used only when there is clear indication because of risk of increased pill burden which in turn could lead to other DRPs.

Adverse drug reactions were also observed in this study. Hypoglycaemia is commonly observed adverse effect among patients receiving oral anti-diabetic drugs or insulin [26]. In this study, a few case of hypoglycaemia is observed and patients should be educated about the sign and symptoms of hypoglycaemia and its management. Antihypertensive agents particularly adverse reactions related to ACEIs were frequently reported and this finding was clearly demonstrated in this study [27]. In such situation angiotensin receptor blockers (ARBs) are recommended however there were no such drugs found in the patients record and this could be attributed to the absence of the drugs in the local market and its cost.

## Conclusion

In the present study, high prevalence of drug-related problems was identified among patients with T2DM and hypertension. The effect of drug treatment being not optimal, untreated indication and symptoms, unnecessary drug-treatment and adverse drug reactions were the most frequent categories of DRPs identified. Therefore, the clinicians should work to improve patient care through prevention and resolving drug related problems since it can affect the quality of the care significantly.

## Limitation

This study used retrospective study design hence it was relying only on information available in the patient records. Laboratory tests used in this study might not be the best methods to monitor blood glucose level. Organ function tests should have been used to determine renal clearance level and hepatic function. In addition, there might be unrecorded ADRs. Therefore, our assessment may not have been comprehensive enough to generate adequate information should generalized cautiously.

## Abbreviations

HFSUH: Hiwot Fana Specialized University Hospital
T2DM: types 2 diabetes mellitus
HNT: hypertension
DRP: drug related problem
ADR: adverse drug reaction
ACEIs: angiotensin converting enzyme inhibitors
CCBs: calcium channel blockers
ADA: American diabetic association
ARBs: angiotensin receptor blockers
